# Surgical Technique: Tscherne-Johnson Extensile Approach for Tibial Plateau Fractures

**DOI:** 10.1007/s11999-013-2962-2

**Published:** 2013-05-14

**Authors:** Eric E. Johnson, Stephen Timon, Chukwunenye Osuji

**Affiliations:** 1 Department of Orthopaedic Surgery, David Geffen School of Medicine, University of California Los Angeles, CHS 76-116, 10833 Le Conte Avenue, Los Angeles, CA 90095 USA; 2 Department of Orthopaedic Surgery, University of Texas, Southwestern Medical Center, Dallas, TX USA; 3 Midwest Orthopaedic Center, Peoria, IL USA

## Abstract

**Background:**

The standard approach to lateral tibial plateau fractures involves elevation of the iliotibial band (IT) and anterior tibialis origin in continuity from Gerdy’s tubercle and metaphyseal flare. We describe an alternative approach to increase lateral plateau joint exposure and maintain iliotibial band insertion to Gerdy’s tubercle.

**Description of Technique:**

The approach entails a partial tenotomy of the anterior half of the IT band leaving the posterior IT band insertion attached to Gerdy’s tubercle. Fracture lines around Gerdy’s tubercle are completed or the tubercle was osteotomized and externally rotated and the joint overdistracted, allowing direct visualization of the joint depression. Joint elevation, grafting, and internal fixation are performed through this window.

**Methods:**

We retrospectively reviewed 76 patients (two groups), Schatzker Types I to II and IV to VI fractures (66 patients), between 1989 and 2005, and 10 patients, with 10 bicondylar posterior plateau fractures, from 2002 to 2010. All patients were followed a minimum of 12 months (average, 3.9 years; range, 12 months to 10 years). Ten patients, with posterior plateau fractures, received anterolateral plateau intraarticular osteotomy for exposure of centroposterior and posterolateral articular depression.

**Results:**

Average knee ROM was 2° of flexion (range, −3° to 5°) to greater than 120° of flexion (range, 100°–145°). In 66 patients, average articular depression improved from 7.4 mm to 1 mm (range, 0–5 mm) and, in 10 posterior fractures, from 18 mm to 1 mm (range, 0–4.5 mm). Infection occurred in one of the 76 patients; acute débridement and intravenous antibiotics resulted in control of the infection.

**Conclusions:**

This approach reliably increases direct visualization of the lateral plateau articular fractures and maintains IT band insertion. Articular osteotomy of the anterolateral plateau provides access to extensive posterior plateau fractures.

**Level of Evidence:**

Level IV, retrospective study. See Guidelines for Authors for a complete description of levels of evidence.

## Introduction

Accepted approaches for tibial plateau fractures vary from minimally invasive to combinations of incisions for complex fracture patterns. Established trends attempt repair of more difficult plateau fractures with minimal exposure and indirect reduction techniques [9, 10, 14, 16, 17]. These techniques reduce the amount of iatrogenic injury to the extremity. Schatzker Types IV to VI [27] and plateaus with metaphyseal comminution or posterior plateau fractures are frequently not amenable to minimal operative exposure [13, 16, 17, 21, 25, 26, 28]. Management of bicondylar plateau fractures generally requires two surgical approaches [2, 5, 6, 13, 18, 32]. Multiple studies have reported approaches for posterior plateau fractures, but these approaches are not extensile to access anterior plateau fractures, meniscal pathology, or metaphyseal-diaphyseal fractures [1, 3, 4, 6–8, 12, 18, 19, 22, 23]. Posterior approaches include posteromedial access [29], posterolateral with fibular osteotomy [12, 18, 22, 25], and prone direct posterior access for shear fractures of the plateau [5, 23] and can produce flexion contractures about the knee [5, 6].

The standard approach to lateral plateau fracture is to elevate the iliotibial (IT) band insertion off Gerdy’s tubercle in continuity with the origin of the anterior tibialis (AT) muscle [1, 27]. The lateral plate fixation is placed on top of Gerdy’s tubercle, and the IT band and AT fascia are then sewn over the plate. This approach is limited to a view of the lateral plateau. It does not allow for extensile exposure for posteromedial, posterocentral, or posterolateral visualization.

The IT band and its insertion into Gerdy’s tubercle help stabilize the anterolateral side of the knee. The IT band is a restraint to medial patellar subluxation; the deep capsule osseous and the superficial layers function as an anterolateral ligament of the knee [24, 26]. It provides posterior pull on the lateral tibial plateau and decreases anterior tibial translation and unloads the anterior cruciate ligament [30].

We describe an approach that differs from the standard anterolateral approach by preservation of the insertion of the IT band attached to Gerdy’s tubercle. Gerdy’s tubercle was osteotomized, if not already a complete fragment and rotated away, from the plateau based on the IT band soft tissue hinge. Temporary transarticular distraction enhances the lateral plateau exposure. Restoration of IT band function to the lateral plateau is achieved by anatomic reduction of Gerdy’s fragment. Plate stabilization is performed on top of the IT band insertion. This approach is extensile and can be used in posterior plateau fractures by incorporating an intraarticular osteotomy of Gerdy’s tubercle, external rotation the tubercle fragment (with its IT band insertion), facilitating exposure of posterior plateau fractures. Using this approach, we report knee ROM, amount of residual articular depression, and overall reduction in preoperative mediolateral tibial condylar width [20].

## Surgical Technique

The indications for this approach were (1) Schatzker Type I to II fractures; (2) Schatzker Type IV fracture with extension of the posterior fracture into the posterolateral plateau; (3) Schatzker Type V to VI fractures; and (4) combined posteromedial shear fractures with posterocentral and posterolateral fractures with intact anterolateral plateau cortex. The contraindication was the classic Schatzker Type IV medial plateau fracture without posterolateral articular fracture extension. Schatzker Type III fractures can be approached with this technique but require an anterolateral Gerdy’s tubercle intraarticular osteotomy extending to the anterior edge of the depressed articular surface to expose the joint depression.

**Fig. 1:**
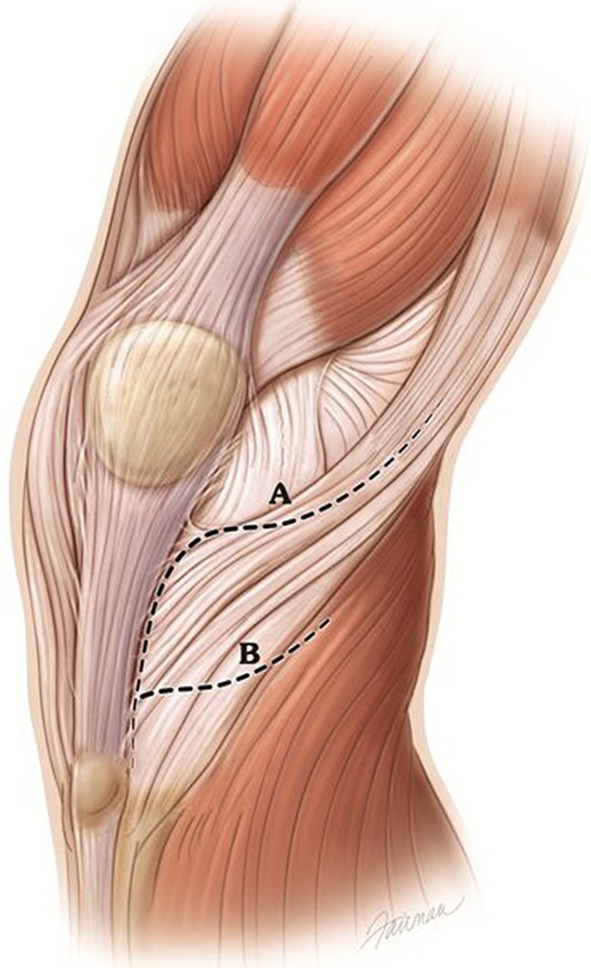
Illustration showing IT band tenotomy, line A, for a left knee. Line B shows level of tenotomy at origin of the anterior tibialis muscle. Capsular and other ligament structures removed for illustration. (Printed with permission from © Fairman Studios LLC)

The patient was positioned supine on a radiolucent table with a bump under the ipsilateral hip. We placed a tourniquet on the thigh and the leg was prepped and exsanguinated. The knee was flexed over a large bump allowing the leg to rest just off the edge of the table. We performed a lateral parapatellar incision from the supracondylar area of the distal femur to below and lateral to the level of the tibial tubercle. A single-layer lateral soft tissue flap is dissected from the wound edge to the posterolateral corner of the tibia. The medial edge of the incision was not elevated over the patellar tubercle unless associated fractures were in this area. We identified Gerdy’s tubercle and the anterior and posterior borders of the IT band. With the knee flexed 40°, the central IT band was incised longitudinally 4 cm above the knee line to where the band crosses the lateral tibial joint (Fig. 1). The incision continues to the lateral joint line, progresses forward proximal to Gerdy’s tubercle to a level lateral to the patellar tendon, and then distally along the lateral edge of the patellar tendon. The anterior half of the IT band at this level is retracted proximally to expose the entire lateral joint line and meniscus attachment. A 2-mm Kirschner wire was placed under the edge of the meniscal coronary ligament to lift up the meniscus facilitating incision of the coronary ligament. We released the coronary ligament from posterior to anterior to the level of the anterior lateral joint line just lateral to the patellar tendon.

**Fig. 2:**
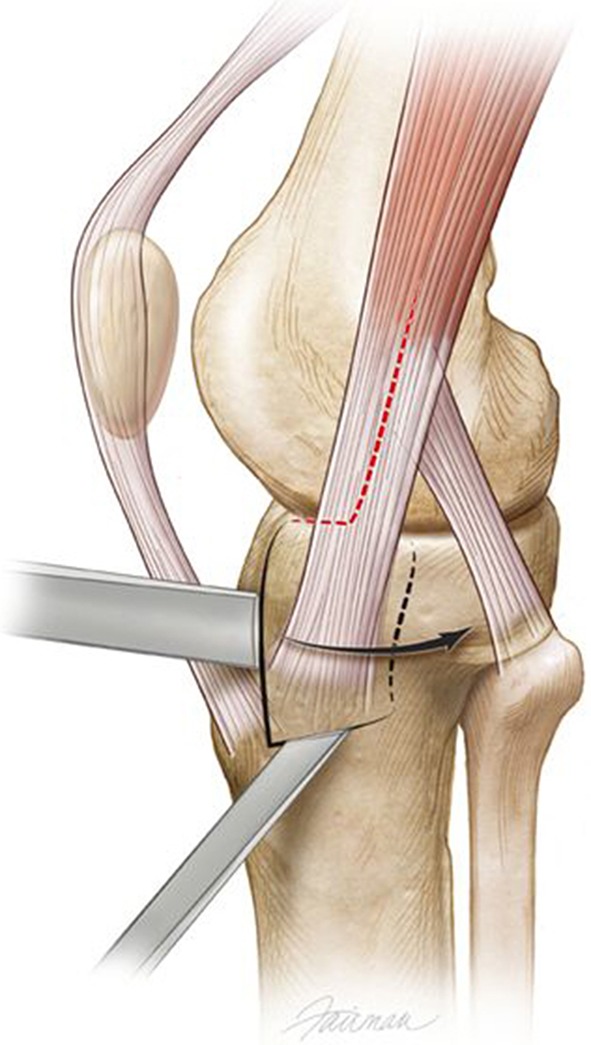
Illustration of lateral view of left knee showing two osteotomes elevating Gerdy’s fragment. Gerdy’s fragment is then hinged outward (direction of black arrow) based on posterior hinge (dotted line behind Gerdy’s tubercle). Associated soft tissues have been removed for illustration. (Printed with permission from © Fairman Studios LLC)

**Fig. 3:**
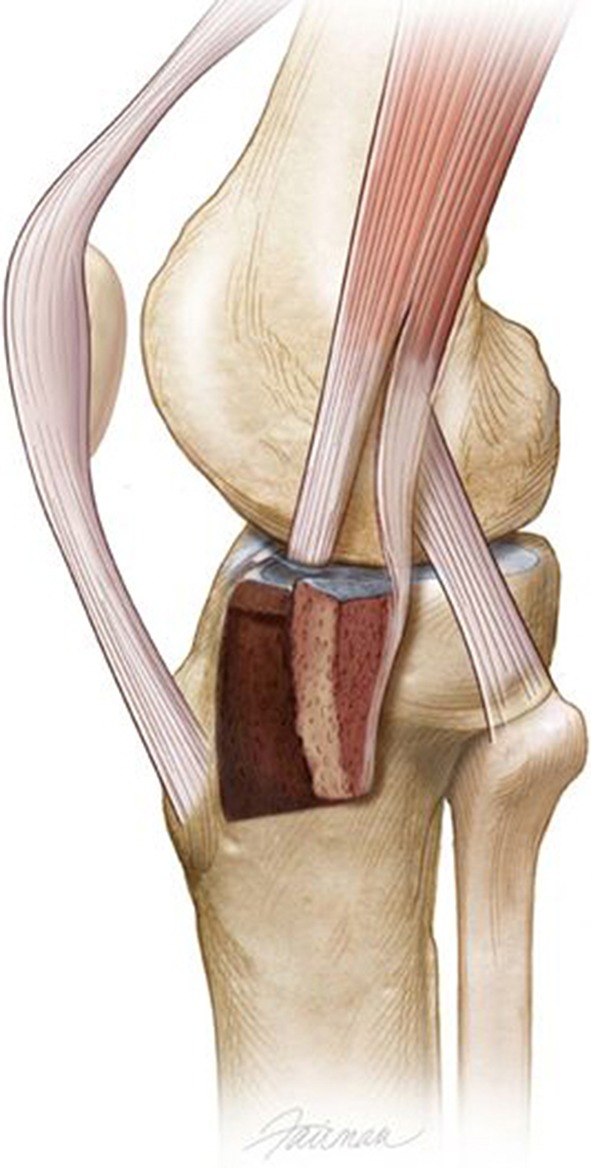
Illustration showing external rotation of Gerdy’s fragment and direct access to the depressed area of the lateral plateau. Posterior insertion of IT band remains attached on the rotated Gerdy’s fragment. (Printed with permission from © Fairman Studios LLC)

The origin of the AT muscle was incised along the lateral tibial metaphyseal flare anterior to posterior to the anterior edge of the proximal tibiofibular joint. Elevation of this muscle distally exposed the lateral plateau in the metaphyseal area. The sagittal fracture line in front of Gerdy’s tubercle is also exposed. We completed any incomplete fracture lines around Gerdy’s fragment with a thin 10-mm chisel (Fig. 2). A lateral-based uniplanar transarticular distractor is placed to apply varus moment to the plateau. External rotation of the Gerdy’s tubercle fragment, on its soft tissue hinge, out of the main plateau, greatly enhances plateau exposure (Fig. 3). Repair of meniscal pathology is performed at this time, before any elevation of depressed articular fragments. We placed three stay sutures of 2-0 absorbable suture in the meniscal edge at positions of posterolateral, midlateral, and anterolateral for final repair of the meniscus to the lateral plateau rim at closure. These sutures provide traction on the meniscus during the lateral joint compartment reconstruction. After elevation and grafting of the articular surface, we reduced Gerdy’s tubercle-posterior IT band to its anatomic position. We positioned lateral locking plates, LCP plates (Synthes, Inc, West Chester, PA, USA), against the lateral shaft and on top of the reduced Gerdy’s tubercle and IT band insertion. Large reduction clamps with ball-tipped spikes, based on opposing intact tibia, provide compression of the plate-plateau interface. A plate compression screw is the first screw placed in the metaphyseal-diaphyseal area of the plate to compress the plate against the bone. We then placed subchondral rafting screws in the proximal portion of the plate. Locking screws were not used in shaft fixation except in patients with osteopenia. Peripheral meniscal attachments were sutured with the three stay sutures to residual coronary ligament tissue on the plateau rim. Closure of the IT band tenotomy and repair of the origin of the AT completed the repair of the lateral soft tissue structures.

**Fig. 4A–J:**
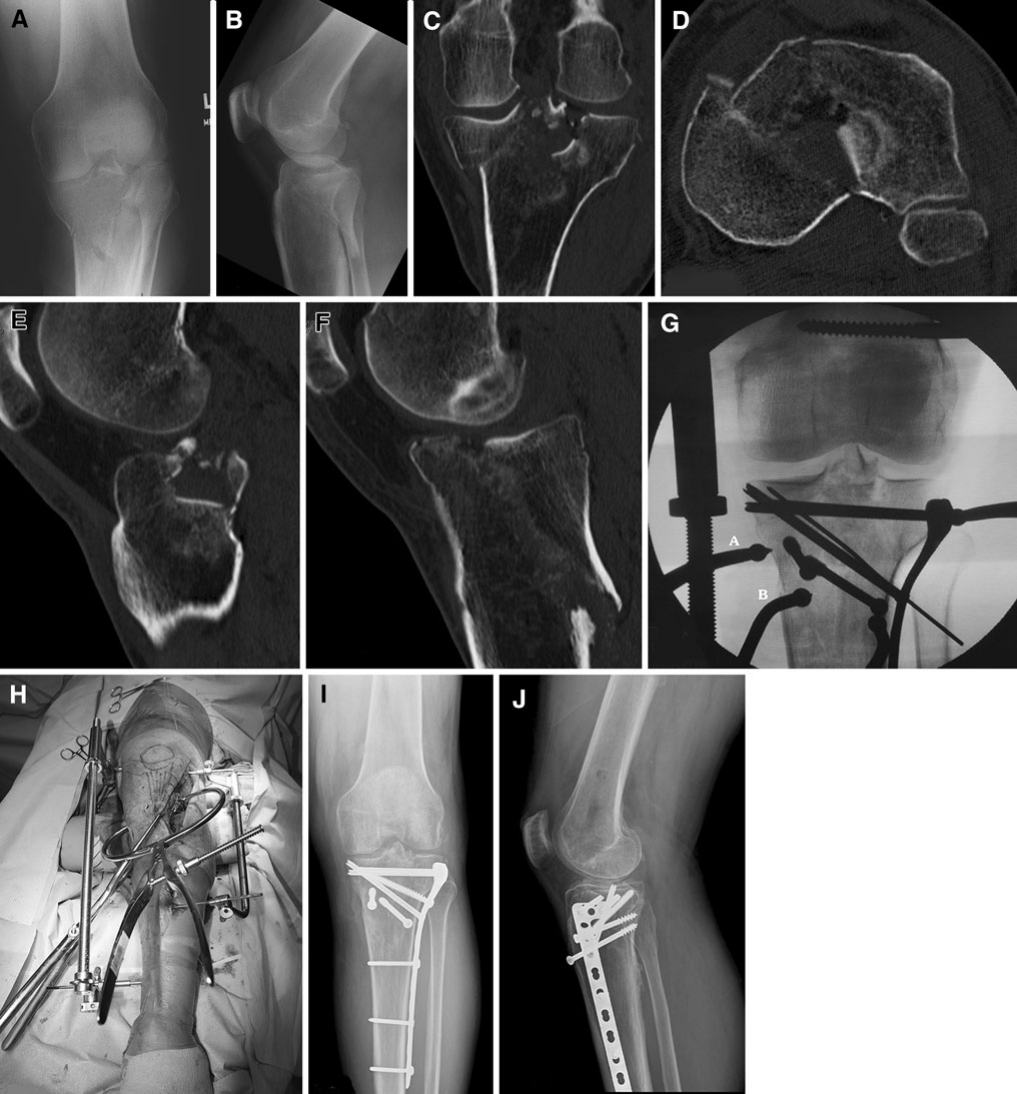
(**A**) Preoperative AP radiograph of a posterior plateau fracture with involvement of posteromedial, posterocentral, and posterolateral left tibial plateau. Anterolateral joint line is intact. (**B**) Preoperative lateral radiograph showing extent of posterior plateau articular displacement and intact anterior plateau cortical bone. (**C**) Coronal CT image showing posteromedial plateau fragment, comminution of tibial eminence, and posterolateral articular fracture depression. Intact anterolateral plateau is present. (**D**) Axial CT image anterior and posterior medial plateau fractures, 90° rotated posterolateral joint fragment, and intact anterolateral cortical margin of the left tibial plateau. (**E**) Centrolateral sagittal CT scan revealing substantial posterior depression of articular cartilage with intact anterior half of plateau cortical bone. (**F**) Sagittal CT scan of medial tibial plateau showing a large posteromedial plateau fragment and oblique major fracture line. (**G**) AP fluoroscopic view of left tibial plateau fracture reduction technique. Ball-tipped clamp A compresses the lateral plate to osteotomized Gerdy’s fragment and lateral plateau bone. Ball-tipped clamp B reduces posteromedial plateau oblique articular fragment through percutaneous insertion. Two separate AP lag screws are placed into the posteromedial plateau. (**H**) Clinical appearance of the use of intraoperative bilateral transarticular coronal plane distractors (medial and lateral), anterolateral incision, and the use of the periarticular tong reduction clamps. (**I**) AP radiographs showing complete healing and axis alignment with anatomic reduction of joint surfaces 3 years after fracture. (**J**) Lateral radiograph of healed left plateau fracture showing reduction of posterior fractures fragments, restoration of plateau joint surfaces, and position of hardware.

Ten patients, with extensive posterior plateau fractures with intact anterolateral tibial plateau cortex, were treated with the same surgical technique using bilateral coronal plane transarticular distractors and an osteotomy of the anterolateral plateau (Fig. 4). Osteotomy planes are the same sagittal (intraarticular, lateral edge of patellar tendon) and axial (metaphyseal) planes at Gerdy’s tubercle (Fig. 2). Rotation of the osteotomized Gerdy’s fragment exposes the centroposterior and posterolateral articular depression. The sequence of fixation of these fractures involves addressing the posteromedial fracture reduction first using a medial-based distractor and either percutaneous reduction clamps with AP lag screws or a separate incision with posteromedial buttress plating for comminuted fracture lines. Depressed central and posterior joint fragments are elevated and grafted through the anterolateral plateau osteotomy site. Closure of the Gerdy’s tubercle osteotomy and lateral-based plate fixation completes the fracture stabilization.

Our protocol uses continuous passive motion machine 2 hours twice a day until knee motion achieves 0° to 95° of flexion. Patients increased knee flexion 5° a day until greater than 90° of painless knee motion was achieved. If patients show lack of progress in knee flexion, knee manipulation is considered 4 to 8 weeks postoperatively. Patients were given walking-assisted aids and instructed to be nonweightbearing for 8 to 10 weeks. Outpatient physical therapy is provided until greater than 120° of knee flexion is achieved. Partial weightbearing was generally allowed after 8 weeks (50 lbs); all patients were reevaluated at 10 weeks to assess joint line depression and fixation integrity. Full weightbearing was achieved by 12 weeks and return to full activities by 16 weeks.

## Patients and Methods

We retrospectively reviewed two groups of patients treated with this approach. The first group of patients included 78 patients with tibial plateau fractures treated between 1989 and 1999 and a second group of 10 patients with extensive posterior plateau fractures treated between 2002 and 2010. All surgery was performed by the senior author (EEJ). In the first group of 78 plateau fractures, seven patients were lost to followup. Three patients were excluded because they had operative procedures at outside institutions. Two were excluded for less than 12 months of followup. Sixty-six patients with lateral plateau combined fractures were left for evaluation. The average age at the time of surgery was 42.5 years (range, 20–81 years). Forty-two patients were male; 24 were female. The minimum was 12 months (mean, 4.1 years; range, 12 months to 10 years).

Fifty-one patients had Schatzker Type I to II fractures and 15 Type V to VI fractures. Thirty-four of these fractures were in the right knee and 32 were the left knee. Preoperative average depression of the articular surface was 7.4 mm (range, 0–15 mm) measured on AP radiographs. Measurements were made from preoperative radiographs between the residual intact articular surface and the largest distance of the subchondral joint line of the depressed fragment. Preoperative mediolateral tibial condylar width was measured from the cortical extent of both medial and lateral plateau edges in the immediate subchondral bone with an average of 87 mm (range, 74–105 mm). All measurements were performed manually from preoperative AP radiographs and CT scans using metric rulers.

Twenty of 66 patients had lateral meniscal avulsion tears and eight patients had bucket handle tears that were repaired. No meniscectomies were performed at surgery in any of these patients. Seven patients had autogenous iliac crest bone grafting, 44 had allograft bank crouton strut grafting, 12 had local bone graft only, and three patients were not grafted. Standard nonlocked plates were used in this group of patients.

Of the second group of 10 patients with extensive fractures of the posterior plateau, all had an anterolateral plateau intraarticular osteotomy and bilateral joint distraction (Fig. 4). There were seven males and three females with an average age of 50 years (range, 34–62 years). All fractures had oblique posteromedial plateau shear fractures combined with posterocentral and posterolateral depression fractures with intact anterolateral plateau cortex. These fractures did not fit the Schatzker classification. These were variants between Types IV and V fractures. There were seven right and three left plateau fractures. Measurements were performed on PACS digital systems using digital rulers on both AP, lateral, and CT radiographs. Preoperative intraarticular fragment depression averaged 18 mm (range, 11–28 mm), and tibial width averaged 95 mm (range, 85–103 mm). These 10 patients had almost 2.5 times the amount of articular depression compared with the first group of 66 patients. Three of 10 patients had additional posteromedial plateau plating for medial cortical comminution. In seven of 10 patients, posteromedial fractures were stabilized with AP lag screws. Bone bank allograft croutons were used as grafting material in all 10 patients.

Patients were seen at intervals of 10 days and 4, 8, 12, and 16 weeks postoperatively. All patients had AP (10° internal rotation view) and lateral radiographs at 30° of knee flexion. Examinations included knee ROM (examined and documented by the senior surgeon), assessment of wound healing, and radiographic assessment of healing by the senior author at each visit. Healing was assessed by progressive remodeling of bone, incorporation of fracture lines, observance of any evidence of loosening of fixation, and maintenance of articular surface reduction. Two authors (EEJ, TS) evaluated all radiographic images within the group of 66 patients, and two authors (EEJ, CO) evaluated all radiographic images of the 10 patients with posterior plateau fractures. Final radiographs were evaluated for amount of depression on both AP and lateral radiographs, incorporation of fracture lines and bone graft, and evidence of any radiolucency or hardware loosening.

## Results

Of the group of 66 patients, the minimum follow-up was 12 months (average 5.6 years; range 12 months–10 years). Average knee ROM was 2° of flexion (range, 0°–5°) to 120° of flexion (range, 100°–145°). Two patients underwent manipulation under anesthesia for ROM that was unsatisfactory at 2 months postoperatively. Each of these two patients had a final followup ROM of least 130° of flexion. Of the 10 patients with osteotomy, the minimum followup was 12 months (average, 28.3 months; range, 12–120 months). Average knee flexion was 0° (range, −3° to 5°) to 128° flexion (range, 100°–145°). In the 66 patients, residual articular depression averaged 1 mm (range, 0–4 mm), and in the 10 patients, residual joint depression average 1 mm (range, 0–4.5 mm). Seven of 10 patients had no joint depression; the other three had 2, 3, and 4.5 mm, respectively.

Mediolateral tibial condylar width was reduced to 79 mm (range, 68–95 mm) in the 66 patients. In the 10 patients with posterior fractures, tibial width was reduced to 83 mm (range, 73–99 mm). Corresponding width of the ipsilateral femoral condyle at the subchondral level for these 10 fractures averaged 79 mm (range, 68–94 mm). By 4 months postoperatively, all 76 patients (both groups) were full weightbearing.

In the group of 66 patients, five patients had hardware removal; two patients older than 70 years of age with preexisting knee arthritis had subsequent TKA 5 and 7 years after injury. There were no postoperative infections in this group of patients. In the group of 10 patients, one patient had a partial lateral meniscectomy for a meniscus tear 27 months after the index procedure. Two patients requested removal of symptomatic hardware at 12 and 17 months from the index operation. There was one perioperative wound infection 3 weeks after surgery. This was managed with débridement and 6 weeks of intravenous antibiotics resulting in complete resolution of infection. There was no failure of fixation and no delayed displacement of the posteromedial plateau fractures.

## Discussion

We describe an approach to tibial plateau fractures that preserves the insertion of the IT band to Gerdy’s tubercle and increases visualization of the depressed lateral plateau. The standard lateral approach to the plateau elevates the insertion of the IT band off Gerdy’s tubercle, is not extensile, and repairs IT band insertion on top of lateral plate fixation. In our technique, plate stabilization is performed on top of the IT band insertion. By the addition of an osteotomy of Gerdy’s tubercle, this exposure becomes extensile for posterior plateau fractures. Our rationale in reporting these two groups of patients is that preservation of IT band anatomy and increased exposure of the lateral plateau with Gerdy’s tubercle rotation enhances exposure and fracture reduction without soft tissue compromise. We also believe that knee ROM is enhanced when a capsulotomy is avoided.

We recognize limitations to our study. First, our cohort was not a homogeneous group. This is a retrospective review of two groups of patients from two different time periods tied together using the same surgical approach. The combination of the two groups, however, shows the advantage of this approach and that extension of the technique to included plateau osteotomy is possible, does not result in loss of knee ROM, or results in loss of restoration of substantial joint depression. Second, the reporting of radiographic measurements of articular residual depression and mediolateral tibial width was accomplished using manual measurement techniques from radiographs of the group of 66 patients. No PACS digital evaluation was available at the time of this evaluation and we have no information on the reliability of the measurement; these should therefore be considered estimates. Third, we did not collect or report patient function at the time of followup and therefore can only report ROM, radiographic findings, and complications.

Previous reports of large extensile exposures have been associated with high rates of arthrofibrosis and increased incidence of infection [1, 13, 14, 28, 32]. We found knee ROM in both series of patients averaged 120° and 128°, respectively. The need for further treatment for postoperative arthrofibrosis and decreased ROM was needed in only two of 66 patients (3%) and none of 10 patients with posterior plateau fractures and osteotomy. Fernandez [11] first reported a series of extensive bicondylar plateau fractures approached through an osteotomy of the patellar tubercle. Our review also shows anterolateral plateau intraarticular osteotomy can provide access to posterior plateau fractures without a patellar tubercle osteotomy or separate posterior incision. We found the articular joint surface elevation was restored to an average of 1 mm of depression. Articular depression less than 2 to 4 mm reportedly does not progress to long-term arthrosis [20]. The logical extension of this approach to manage posterior plateau fractures became evident with the extent of the exposure obtained by outward rotation of Gerdy’s fragment (Fig. 3). The initial use of anterolateral intraarticular plateau osteotomy was, in 2002, to treat an extensive posterior plateau and shaft fracture. No single approach was available to manage the posterior plateau and metaphyseal-diaphyseal fractures of the shaft with an intact anterolateral tibial plateau. A key requirement for anterolateral plateau osteotomy is that posterior articular fracture extension must enter the posterolateral plateau for the osteotomy to visualize the depressed posterocentral plateau fractures.

Posterior plateau fractures are less frequent and generally require a separate posterior approach [5, 7, 18, 19, 23]. Tao et al. [23] described an L-shaped posterolateral approach with direct access to the plateau between the lateral gastrosoleus muscle and posterior tibial neurovascular bundle. Several authors have described a posterolateral plateau approach combined with a proximal fibular osteotomy [12, 18, 22, 25]. Although these approaches are directed at posterior fracture patterns, they are not extensile to access anterior plateau pathology. Yoo et al. [31] found a higher load to failure of simulated posteromedial fractures stabilized with double plate fixation of the posteromedial plateau. In contrast, Higgins et al. [15] found no difference in final load to failure of single lateral locked plate fixation of simulated bicondylar fractures compared with double plating but less subsidence with double plating. With our extensile approach, exposure and fixation of both lateral component plateau fractures and posteromedial, posterocentral, and posterolateral fragments is possible and may not require a second approach for posteromedial plating.
